# Hentges, Gudrun, Georg Gläser, und Julia Lingenfelder (Hrsg.) (2021): *Demokratie im Zeichen von Corona*: Berlin: Metropol Verlag. 370 Seiten. 26,00 €

**DOI:** 10.1007/s11615-023-00459-1

**Published:** 2023-03-28

**Authors:** Klaus Moegling

**Affiliations:** Universität Kassel, i. R., Kassel, Deutschland

Die Einleitung der Herausgeber:innen gibt zunächst einen Überblick über interdisziplinäre und insbesondere gesellschaftswissenschaftliche Studien zur Corona-Problematik, um in diesem Kontext die Fragestellung des Bandes zu stellen: „In diesem Band wollen wir aus sozialwissenschaftlicher Perspektive analysieren, in welcher Weise sich die Corona-Pandemie auf Staat, Gesellschaft, Ökonomie ausgewirkt hat bzw. auswirken wird“ (S. 11). Hierbei solle der bisher vernachlässigte Zusammenhang zwischen der Corona-Pandemie und der Demokratie-Entwicklung im Mittelpunkt stehen. Die Herausgeber:innen entscheiden sich hierbei für das Verständnis einer sozialen Demokratie, die über die politische bzw. liberale Demokratie hinausgeht und das Verhältnis von Kapitalismus und Demokratie reflektiert. Daher geht es ihnen nicht nur um die Frage, ob staatliche Maßnahmen hinsichtlich der Corona-Pandemie in Übereinstimmung mit den Grundrechten im Sinne der Abwehr gegen staatliche Willkür stehen, sondern auch darum, welchen Einfluss sie auf die Frage sozialer Ungleichheit bzw. auf die soziale Krise der Demokratie haben (S. 22–23).

Dementsprechend folgern die Herausgeber:innen auch im Rahmen der Kurzvorstellung der verschiedenen Beiträge des Bandes zusammenfassend und den Beiträgen vorgreifend: „Der Coronavirus kann potenziell jede und jeden treffen, doch nicht alle sind gleich betroffen. Dies ist sehr schnell in Bezug auf die Risikogruppen deutlich geworden. Jedoch vollziehen sich die Betroffenheiten, die sich aus der Pandemie, staatlichen Maßnahmen, zivilgesellschaftlichen Reaktionen und wirtschaftlichen Folgen ergeben, darüber hinaus an bestehenden Spaltungslinien wie ‚race‘, class, gender etc.“ (S. 25).

Der in dieser Einleitung angesprochene komplexere Blick auf den Zusammenhang von Demokratie und Corona ist eine Stärke des Herausgeberbandes, der auch in der Auswahl und Bearbeitung der weiteren Beiträge deutlich wird.

Der Einleitung folgt zunächst ein Beitrag von *Klaus Dörre*, der die Corona-Krise vor dem Hintergrund der derzeitigen ökonomisch-ökologischen Krise analysiert. Hierbei laufe es letztendlich darauf hinaus, entweder konsequent in ein sozialökologisches Transformationszeitalter einzutreten oder aber „durch Ökozid, einen verheerenden Atomschlag oder einer außer Kontrolle geratenen Pandemie“ (S. 64) das Anthropozän zu beenden.

Der dritte Beitrag des Bandes wurde von *David Salomon* verfasst und thematisiert kenntnisreich die Frage des entgrenzten Ausnahmezustands in der Corona-Pandemie, bei dem der Ausnahmezustand möglicherweise zur Regel werde. Insbesondere betont er die Notwendigkeit zum Erhalt bzw. zum Ermöglichen einer kritischen Öffentlichkeit auch in Pandemie-Zeiten, selbst wenn die Kommunikation in Präsenzformaten nicht möglich ist.

Unter dem Aspekt der sozialen Ungleichheiten versammeln sich anschließend verschiedene Beiträge, die sich auf bestimmte soziale Gruppierungen beziehen und deren Betroffenheit durch die Pandemie und den gesellschaftlichen Umgang hiermit analysieren und beurteilen. *Christoph Butterwegge* schreibt über die wachsende Kluft zwischen Arm und Reich in der Pandemie. *Michael Klundt* bezieht sich auf die unterschiedliche Betroffenheit der Generationen. *Hannah Quinz, Johanna Neuhauser* und *Jörg Flecker* beziehen sich auf die Wahrnehmung sozialer Ungleichheit in der österreichischen Bevölkerung während der Pandemie. *Alexandra Scheele* thematisiert die geschlechtsspezifische Betroffenheit von Männern und Frauen unter Corona-Bedingungen. *Wiebke Judith* schreibt über die gesellschaftliche Situation von Geflüchteten während der Corona-Pandemie. Alle diese Beiträge sind dem erweiterten Blick einer sozialen Demokratie verpflichtet und gehen deutlich über eine formaldemokratische Analyse im Zusammenhang mit Corona hinaus.

Im Rahmen des folgenden Kapitels „Extreme und politische Rechte“ beginnen *Gudrun Hentges* und *Kemal Bozay* mit einem Aufsatz über „Hate Speech“, „Fake News“ und „Verschwörungsfantasien“. *Gudrun Hentges *und *Gerd Wiegel *setzen dieses Kapitel mit Ausführungen über die Instrumentalisierung der Corona-Pandemie durch Rechtsextreme fort. *Daniel Keil* bezieht die Thematik auf die europäische Rechte.* Georg Gläser *versteht die Aktionen der Trump-Administration im Zuge der Corona-Pandemie als Ausdruck eines Autoritären Populismus. *Magdalena Marsovszky* befasst sich mit dem ungarischen Notstandsgesetz zur Eindämmung der Corona-Pandemie. Im Rahmen dieses Kapitels werden u. a. sorgfältige Analysen verschiedener rechter Gruppen und Bewegungen mit der Rekonstruktion ihrer Verschwörungsmythen verbunden.

Das Kapitel „Zivilgesellschaftlicher Protest“ bezieht sich auf Aktionsformen in Deutschland und Israel. Hier findet sich ein englischsprachiger Beitrag von *Sophia Solomon, Jakob Andrae* und *Felix Kirchhof* zum Vergleich deutscher und israelischer Proteste gegen die staatliche Corona-Politik. Wichtig ist hierbei auch der Bezug zu Formen der sog. Geschichtsklitterung, wenn angesprochen wird, dass sich Protestierende gegen die staatlichen Corona-Maßnahmen auch mit KZ-Kleidung anzogen oder andere einen gelben Stern trugen (S. 289).

*Julia Lingenfelder *vergleicht das Potenzial von Aktionsformen im Zuge der Corona- und der Klimakrise und stellt einen Nachteil sozialökologischer Bewegungen gegenüber den sich über staatliche Regeln hinwegsetzenden Corona-Protesten fest, welche die mediale Öffentlichkeit auf sich ziehen können.

Im abschließenden Kapitel „Politische Bildung und Soziale Arbeit“ analysieren *Kemal Bozay *und *Burak Çopur* das demokratiefeindliche Potenzial der Corona-Proteste und verweisen auf Aspekte der sozialen Arbeit und politischen Bildung, um hier präventiv gegenzusteuern. In einem zweiten Beitrag entwickelt *Klaus-Peter Hufer *die Notwendigkeit, auch in Corona-Zeiten sich auf das Wesen politischer Bildung zu besinnen. Hierbei befasst er sich mit dem Widerspruch zwischen Mündigkeit und Verschwörungsfantasien und wie politische Bildung dies zum Thema machen könne. Abschließend befasst sich *Alexander Wohnig* ebenfalls mit den Möglichkeiten politischer Bildung, um den gesellschaftlichen Umgang mit der Pandemie zu thematisieren. Hierbei gehe es nicht primär um die Erzeugung eines an die Pandemie angepassten Verhaltens, sondern vor allem um die Analyse und Beurteilung politischer, ökonomischer und gesellschaftlicher Strukturen, die im Umgang mit Covid-19 erkennbar werden. Gerade dieses letzte Kapitel über die politische Bildung in den verschiedenen gesellschaftlichen Bereichen ermöglicht dann auch, dass der_die Lesende nicht pessimistisch zurückgelassen wird, sondern sich eine Perspektive präventiver Bildungsarbeit eröffnet, die eine Anfälligkeit gegenüber Verschwörungsmythen verringern und die Bereitschaft, für eine soziale Demokratie einzustehen, erhöhen könnte.

Insgesamt liegt ein Herausgeberband vor, der den gesellschaftlichen Umgang mit der Corona-Pandemie mehrperspektivisch und vor dem Hintergrund gesellschaftstheoretisch verankerter Analysen thematisiert. Der Fokus auf den Zusammenhang von Pandemie und Demokratie bietet einen wichtigen Einblick in vergangene und aktuelle politische Auseinandersetzungen, die im vorliegenden Band sorgfältig ideologiekritisch untersucht werden. Der Band ist sehr geeignet als Grundlage politikwissenschaftlicher Seminare, die sich insbesondere mit staatlichen Regulierungsversuchen von Krisenphänomenen und deren gesellschaftlicher Resonanz auseinandersetzen.

